# More Information = Less Aggression? Impact of Information Asymmetry on Chinese Patients' Aggression

**DOI:** 10.3389/fpubh.2019.00118

**Published:** 2019-05-22

**Authors:** Yuepei Xu, Wen He

**Affiliations:** Department of Psychology, Shanghai Normal University, Shanghai, China

**Keywords:** information sharing, aggression, trust, doctor–patient relations, information asymmetry, mediate effect

## Abstract

**Background:** Information asymmetry is a widely studied economic phenomenon. It refers to the situation in which one group in a transaction has more information than the other. Nowadays, information asymmetry has been studied not only as a financial topic but also as a potential reason for essential social problems.

**Objective:** To take Chinese doctor–patient relationship as an example and investigate the relationship among information asymmetry, trust level, and aggression behavior using an experimental design.

**Methods:** A total of 44 undergraduates (information asymmetry group, *N* = 22, 5 males, 17 females, mean age = 18.95, *SD* = 0.18; information symmetry group, *N* = 22, 7 males, 15 females, mean age = 19.27, *SD* = 0.18) took part in our experiment. Different slides and guidance were used to create a virtual information asymmetry situation, and we use the Wake Forest Physician Trust Scale (WFPTS) and the hot sauce allocation paradigm to measure their trust level and aggression, respectively.

**Results:** Participants in the information asymmetry group allocated significantly more hot sauce to the doctor (*p* <.005, *d* = 1.09) and displayed significantly lower trust level (*p* < 0.05, *d* = −0.78) than the control group. Patients' trust level had a significant mediating effect (95% confidence interval [−1.39, –0.05]).

**Conclusion:** Asymmetric information may arouse patients' aggression and lower their trust in doctors. Patients' trust level is also a significant partial mediator between their aggression and information asymmetry. The current study reinforces the urgent need for information openness in the Chinese medical system.

## Introduction

Information asymmetry (also known as information incommensurateness) is a widely studied economic phenomenon. It is defined as the situation in which one group in a transaction or communication has more information (usually better) than the other (1). As early as the 1970s, researchers have pointed out the serious results of information asymmetry, which is the breakdown in the functioning of the capital market (2). Nowadays, information asymmetry has been studied not only as a financial topic but also as a potential reason for essential social problems, such as weak doctor–patient relationship (DPR) [see (3, 4)] and trust crisis among different social groups [see (5)].

As a source of communication, information is usually known as the resolution of uncertainty (6). However, in the context of information asymmetry, information obviously does not fulfill its mission of eliminating uncertainty. Ironically, asymmetric information worsens uncertainty for one party while allowing the other party to dominate. This kind of uncertainty may lead to a series of negative results, including a lower trust level of the inferior group toward the dominant group and potentially increased aggression from the inferior group.

## Impact of Information Asymmetry on Trust and Aggression

According to the path model of specific trust (7), when trust behavior is used to rely on a stranger, we tend to fully mobilize our psychological resources, and our perceived trustworthiness to the trusted target plays an essential role. As early as 1999, researchers had suggested that the openness of a system or information can create a climate of trustworthiness (8). With asymmetric information, especially when the inferior group may be aware that some information is not available to them, the level of trustworthiness perceived by the inferior group is significantly lower regardless of whether the other group is trustworthy. As such, the group suffering from asymmetric information may not be willing to trust the other group. This effect is why information symmetry is particularly important in relationships that require a high level of trust, such as DPR (9, 10).

Moreover, asymmetry information usually means uncertainty, which may lead to increased aggression. It should be noted that people appear to naturally hate and cannot stand uncertainty (11, 12). Several studies have shown that the level of uncertainty can negatively affect an individual's judgment and behavior (13, 14). Due to individuals' intolerance of uncertainty, they may take actions to reduce or avoid uncertainty of the environment. In real life, uncertainty cannot be avoided, and we have to communicate or transact with dominant groups under information asymmetry. Under high-level uncertainty, individuals' cognitive ability may be damaged, and their emotion may be unstable (12, 15). Even worse, many researchers suggested that with increased asymmetry information, individuals may perceive information as threatening, which leads to a series of stress reactions including rapid heartbeat and high blood pressure (16). As such, under information asymmetry, individuals' damaged cognitive ability leads to biased information processing, causing them to consider the environment as threatening. Moreover, individuals produce more stress reactions physiologically. Both of these possible effects may cause individuals under information asymmetry to become aggressive. What's more, a study also suggested that information asymmetry allowed the community of interest to be destroyed and resulted in the inferior group having a feeling of imbalance, which may cause some exclusive offensive behavior (10). Based on the above, in this current study, we assumed the following:

H_1_: Information asymmetry may increase individuals' aggression behavior and decrease the trust level.

## Trust Level as a Mediator

Trust level appears to have a negative effect on an individual's aggression. Previous studies suggested that the higher the individual's trust level in the external environment and other individuals, the lower their aggression is; that is, a significant negative correlation occurs between an individual's trust level and aggression behavior (17). Studies also suggested that a low level of trust can lead directly to high and stable aggression (18). As such, as a factor decreasing trust level, information asymmetry may have an indirect effect on aggression through trust level. In this current study, we hypothesized the following:

H_2_: Trust level may negatively predict patients' aggression and be a significant mediator between aggression and information asymmetry.

## Using Chinese Doctor–Patient Relationship as an Example

Most studies on information asymmetry are analytical studies rather than empirical ones. The psychological effect of information asymmetry is also not clear. Therefore, the current study tends to use Chinese DPR as an example to investigate the relationship among information asymmetry, trust, and aggressive behavior.

The relationship between Chinese doctors and patients is a classic example of the information asymmetry relationship. In China, researchers have suggested that information asymmetry may be one of the main causes of an increasingly poor DPR [see (4, 19)]. Patients' lack of medical knowledge may place them at a disadvantaged position when deciding on their medical treatment (20). Some studies also pointed out that with the advantage of more information, hospitals may provide overloaded medical service in order to pursue more benefits (10, 21). In most Western countries, DPR has experienced a great evolution (22). The patient's role has changed from simply obeying the doctor like a child to seeking mutual participation like an adult (23). Nowadays, more researchers consider “patient-centeredness” essential to DPR (24). However, because of cultural specificity and different medical systems, the evolution of Chinese DPR seems to be much slower (25), and the so-called “patient-centered medicine” is still far from being realized in China. Most of the time, because of information asymmetry, doctors may simply consider patients as laymen without any medical knowledge, which causes patients to lose their subjectivity. The significant result is that the relationship between doctors and patients gradually becomes indifferent, which leads to a crisis of trust. Despite flaws, we admit that the relationship between doctors and patients in China is still similar to the relationship between adults and children, or worse. As such, in the communication between Chinese doctors and patients, patients should be the inferior party, and the doctor should be the dominant party.

Therefore, our two hypotheses can be rewritten as follows:

H_3_: Information asymmetry may increase patients' aggression behavior against doctors and decrease their trust level in doctors.

H_4_: Patients' trust level tends to negatively predict aggressive behavior and is a significant mediator between patients' aggression and information asymmetry.

## Method

### Participants and Design

All participants were university students, most of whom were undergraduates (not psychology majors) at a university in Shanghai (*N* = 44, 12 males, 32 females; mean age = 19.11, *SD* = 0.84). The current study was a single-factor between-subject design. The only independent variable is information symmetry, whereas the dependent variable is patients' aggression, and the mediator is patients' trust in doctors. Participants were randomly allocated, within gender, to the experimental group (information asymmetry, *N* = 22, 5 males, 17 females; mean age = 18.95, *SD* = 0.18) or the control group (information symmetry, *N* = 22, 7 males, 15 females; mean age = 19.27, *SD* = 0.18). All participants were given a small gift after the experiment, and we disclosed the experiment to them as well.

### Materials and Measures

#### Wake Forest Physician Trust Scale

The WFPTS (26) is a widely used 10-item scale to rate patients' trust in doctors based on the US medical system. The current study used the Chinese version of the WFPTS. Due to the different medical systems between China and the US, the Chinese version changed some items in the original scale. The Chinese WFPTS has 10 items with two dimensions, benevolence and technical competence, and each dimension consists of five items. Studies have shown that the Chinese WFPTS had good reliability and validity (α = 0.89, test–retest reliability was 0.83), which could be used as a tool for measuring Chinese patients' trust (27, 28).

#### Hot Sauce Allocation Paradigm

The current study aimed at investigating patients' aggression toward doctors instead of trait aggression level. As such, using any aggression scale was not appropriate. The hot sauce allocation paradigm provides a new way to measure individuals' aggressive behavior by creating a specific situation in which participants perceive the potential for real harm to come to the target (29). Participants were told that the target hated spicy food, and then they were asked to allocate hot sauce for the target. The grams of hot sauce can be used as the level of participants' aggression toward the target. Many later studies have used it successfully; for its reliability and validity, see Ritter and Eslea (30).

## Procedure

To increase participants' degree of involvement, our laboratory was decorated like a real hospital (including labeling our room as “Clinic Room,” posting Red Cross signs, and so on). We also asked a middle-aged man who was actually one of the staff members of the university to act as a fictitious doctor. During the entire experiment, experimenters called the middle-aged man “Doctor Wang.”

In the beginning, the participant and the doctor were asked to stay in the clinic room together for a while. Then, the doctor left the room, and we let the participant imagine that he caught a cold and had to go to Doctor Wang for medical treatment. Before going to Doctor Wang, participants were asked to fill in the medical record card (we collected age and gender from this card). Afterwards, we told participants that the hospital they visited provided a slide (see slides in our Supplementary Materials) about their disease, so they may read the slide carefully to review it.

The slides we provided were different for the experimental group and control group, and all information in the slide was fictitious. In the experimental group (information asymmetry group), sensitive information (e.g., the profit of medicine) in the slide was hidden and labeled as “Sorry, this information is only available for doctors,” whereas in the control group (information symmetry), all information was available. After reading the slide, we also reminded the participants that some information was not available for patients but only for the doctors. Then, participants were asked to fill in the WFPTS.

Next, we told participants that the experiment was finished, but a different researcher was doing another study about individual taste preference at the same time. Participants were asked to fill in a taste preference scale that we were not interested in. Then, we gave the fictitious Doctor Wang's taste preference scale, which clearly showed that Doctor Wang strongly disliked spiciness. Lastly, the participants were asked to make a sandwich for Doctor Wang using the hot sauce we provided. The grams of hot sauce were recorded keeping one decimal.

## Data Analysis

All data were recorded and processed using SPSS Statistics 19.0 and JASP 8.6.0. For comparison of the aggression and trust level between the information asymmetry and information symmetry group, we used independent *t* test and calculated Cohen's *d* and its 95% confidence interval (CI) as the effect size. For mediation analyses, we used the PROCESS macro (31).

## Results

After the experiment, we excluded two participants because one participant's *Z* score of aggression is over 3.0 and another participant had misunderstood our instructions (she thought she would eat the sandwich).

**Figure 1:**
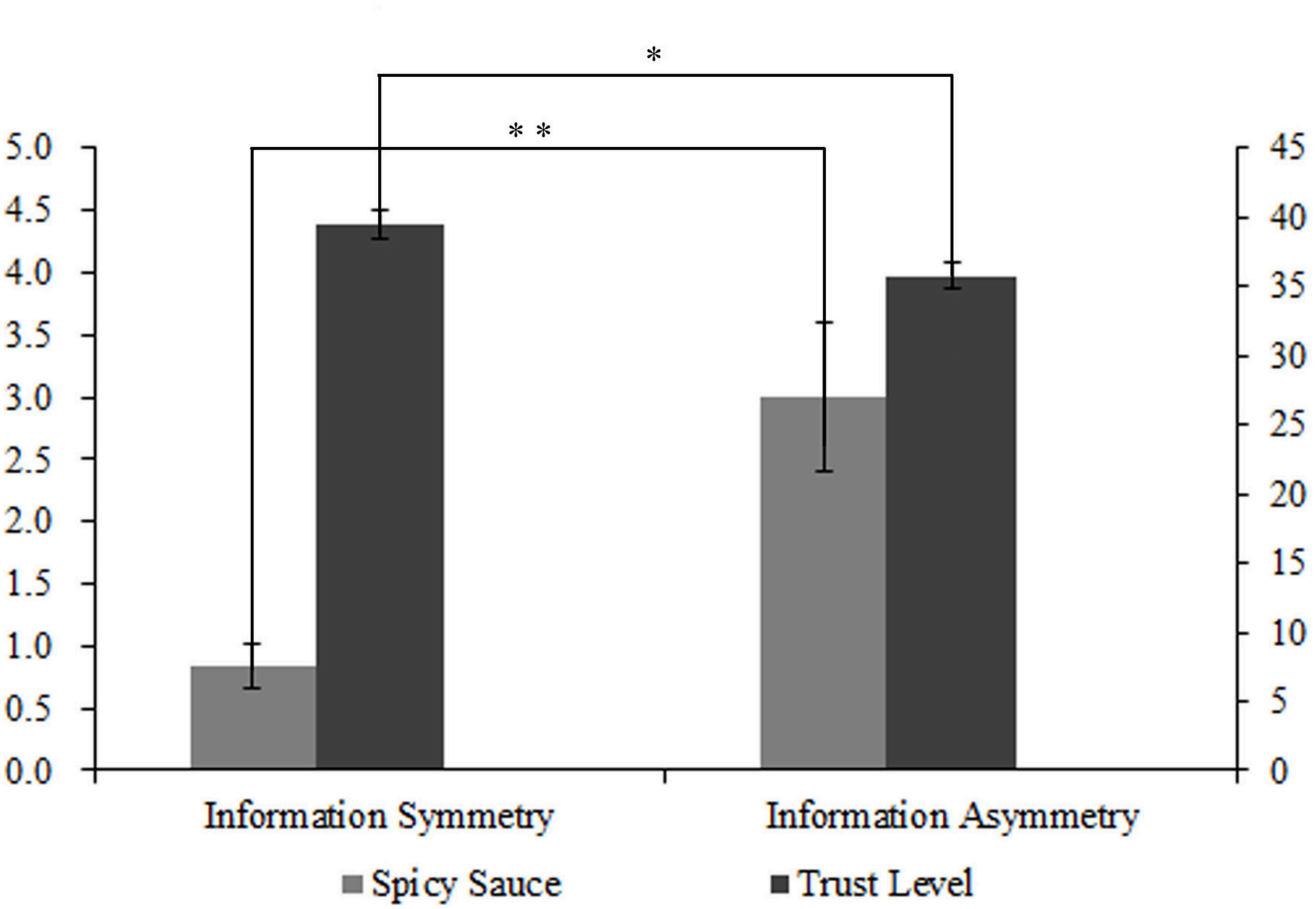
Mean grams of spicy sauce and mean trust level from different information symmetry groups. **p < 0.005, *p <0.05.

As shown as Figure 1, the data showed that the participants in the information asymmetry group (*M* = 3.00, *SD* = 2.70) allocated significantly more hot sauce for Doctor Wang than the information symmetry group did (*M* = 0.83, *SD* = 0.81), *t*_(40)_ = 3.53, *p* = 0.002, Cohen's *d* = 1.09, 95% CI (0.43, 1.73). Moreover, participants in the information asymmetry group (*M* = 35.71, *SD* = 4.37) showed significantly less trust in Doctor Wang (*M* = 39.48, *SD* = 5.19), *t*_(40)_ = −2.54, *p* = 0.015, Cohen's *d* = −0.78, 95% CI (−1.41, −0.15).

**Figure 2:**
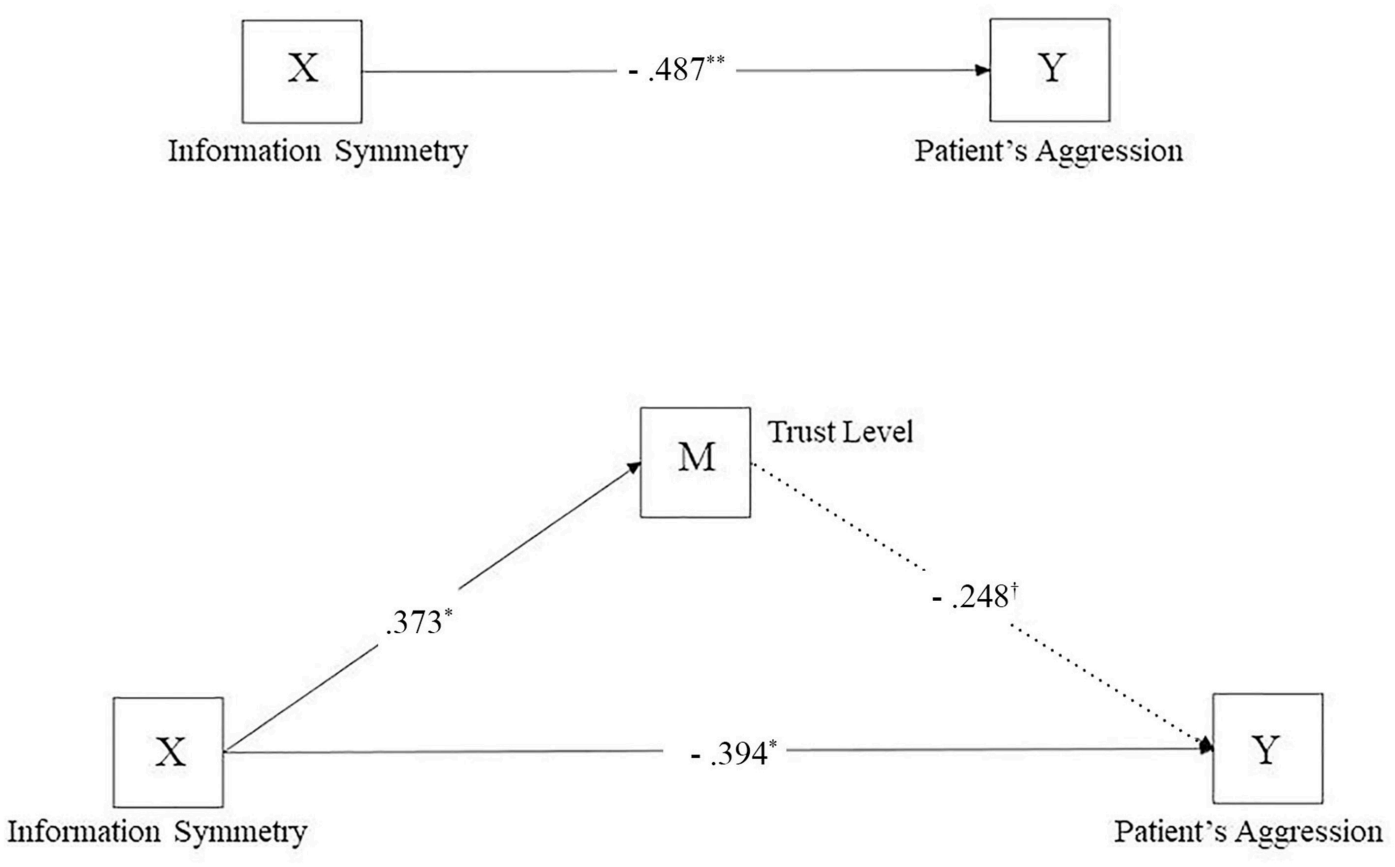
Path diagram of the meditate effect of trust level between information symmetry and patient's aggression. All coefficients are standardized; **p < 0.005, *p < 0. 05, ^†^*p* < 0.1.

We also found that participants' trust in doctors was negatively correlated with their aggression to doctors. In other words, the higher trust level is, the lower the aggression (Pearson's *r* = – = 40, *p* = 0.01). For mediation analyses, we chose 5,000 samples for bias-corrected bootstrap confidence intervals. If the 95% confidence interval does not contain 0, the mediated effect is statistically significant (31). The result showed that trust level was a significant mediator between information symmetry and patients' aggression, with a 95% confidence interval (−1.39, −0.05), and the effect size was −0.41 (see Figure 2 for path diagram). Moreover, after controlling the trust level, the direct effect of information symmetry to patients' aggression was still significant, with a 95% confidence interval (−3.41, −0.92), and thus, the trust level partially mediated the effect of information symmetry on patients' aggression.

## Discussion

The result supports our hypothesis that asymmetric information arouses patients' aggression and lowers their trust in doctors. Even more interestingly, patients' trust level is a significant partial mediator between patients' aggression and information asymmetry. As individuals' trust level is a partial mediator, other mediators (e.g., sentiment) between aggression and the effect of information asymmetry may have not been found, which reminds us that further study should investigate this topic systematically. In addition, more empirical studies on information asymmetry and individuals' psychological process are needed.

The current study first gave a preliminary conclusion of the impact of information asymmetry on individuals' psychology (lower trust) and behavior (more aggression), giving evidence that asymmetric information can cause not only economic chaos but also social problems (e.g., worse DPR). Our results encourage more studies on behavioral and psychological consequences of information asymmetry instead of purely economic studies.

Multiple negatively psychological consequences are caused by information asymmetry. First, because of unknown information, perceived uncertainty may significantly increase. Uncertainty causes individuals to lose cognitive abilities and produce more stress reactions (12, 16, 32). Moreover, asymmetric information causes individuals to feel that they are being cheated upon, resulting in lower perceived trustworthiness. Most often, the other side of the transaction is a stranger to an individual, just like a doctor to a patient in this current study. Without understanding each other, lower perceived trustworthiness is fatal to individuals' trust level. Conversely, we also believe that sharing information can increase individuals' perceived trustworthiness, as well as individuals' trust level to their target. It is a simple logic similar to “you tell me more, so I trust you more,” whereas hiding information always leads to untrustworthiness.

Interestingly, trust level is a significant partial mediator, proving that lower trust level also predicts more aggression to some extent. However, we believe that a more complex relationship or even more factors play a role. For example, as mentioned above, emotion may impact individuals' aggression, and some studies showed that negative emotion may decrease individuals' trust level (33). As such, further studies should build a more complete model describing the relationship among all these factors, and more mediators between information asymmetry and patient's aggression need to be explored.

Although the current study was merely a preliminary primer on this topic, we proposed a practical paradigm, which is the virtual situation creation using slides and guidance. We believe that this method, using a fictitious doctor and asking participants to imagine they are going to ask for treatment from the doctor, may be used in further studies to investigate DPR and information asymmetry in all cultural backgrounds using an experimental and empirical approach.

Finally, some limitations of this study should be pointed out. Issues on reliability and validity of the first paradigm require further discussion. For example, despite our best effort to increase the involvement of our participants, a main difference between participants and real patients is that the participants are all free from physical pain, whereas real patients may be suffering from a variety of symptoms. What's more, all of our participants are college students and the sample size was small to some extent, which reminds us that the homogeneity of participants is high and the current study lacks more participants from different ages and occupations. It is possible that patients under pain and symptoms have a more acute response to asymmetric information, which means that aggression and decrease in trust may be underestimated.

## Conclusion

Asymmetric information may arouse patients' aggression and lower their trust in doctors. Moreover, patients' trust level is a significant partial mediator between patients' aggression and information asymmetry. The current study reinforces the urgent need for information openness in the Chinese medical system.

## Data Availability

All datasets generated for this study are included in the manuscript and/or the Supplementary Files.

## Ethics Statement

This study was approved by the Shanghai Normal University Academic Ethics Committee. All subjects gave written informed consent in accordance with the Declaration of Helsinki.

## Author Contributions

WH presented the idea of this article, participated in the discussion of the experiment, guided and modified the draft. YX collected the data and wrote the draft.

### Conflict of Interest Statement

The authors declare that the research was conducted in the absence of any commercial or financial relationships that could be construed as a potential conflict of interest.
