# Supplementary material for: More Information = Less Aggression? Impact of Information Asymmetry on Chinese Patients' Aggression
Source: Front Public Health. 2019 May 22;7:118. doi: 10.3389/fpubh.2019.00118 (PMC6540841; doi:10.3389/fpubh.2019.00118)
Supplement: Supplementary file 2 [file Presentation_1.pptx]

## Slide 1
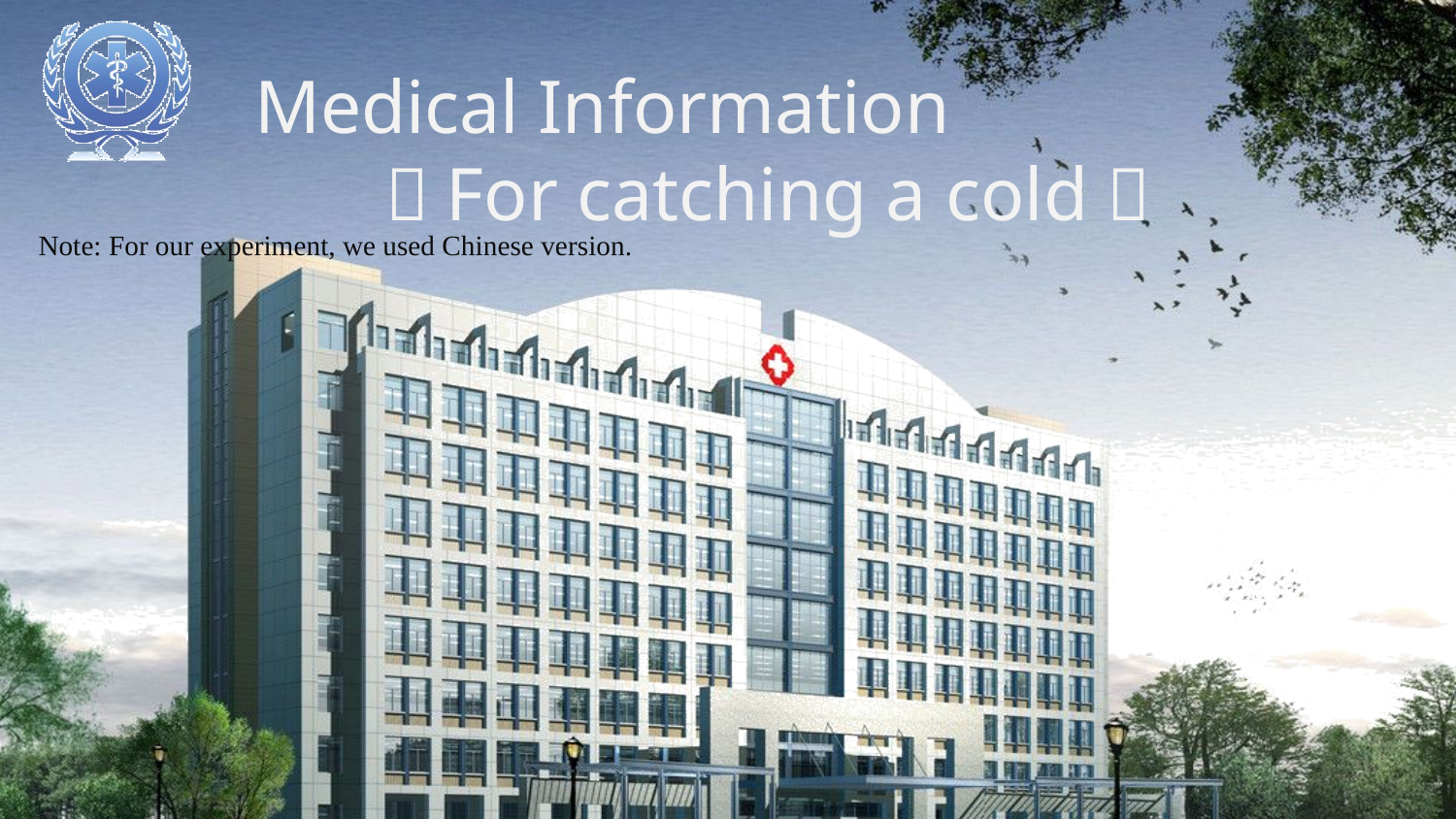

Medical Information
 （For catching a cold）
Note: For our experiment, we used Chinese version.

## Slide 2
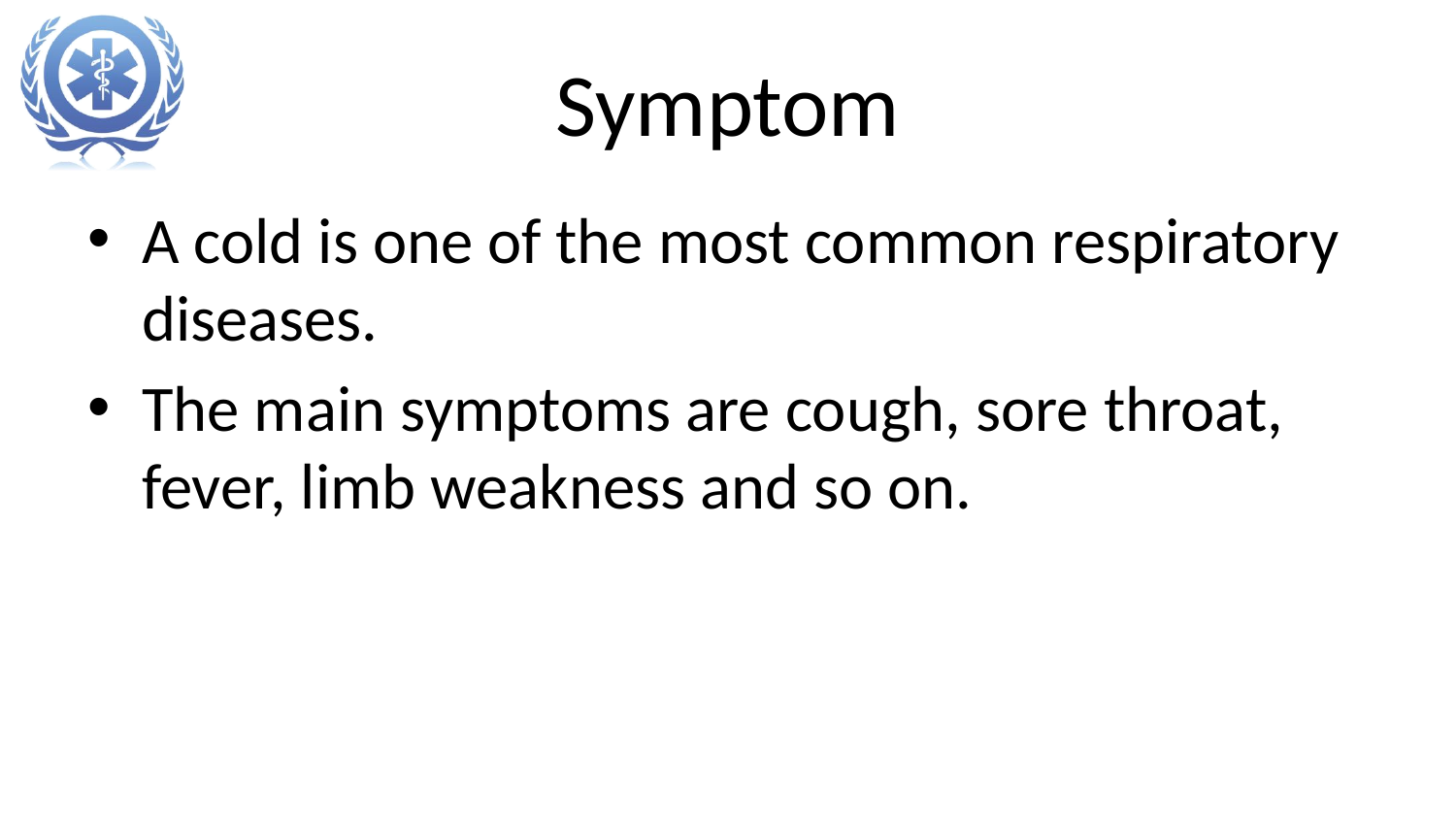

# Symptom
A cold is one of the most common respiratory diseases.
The main symptoms are cough, sore throat, fever, limb weakness and so on.

## Slide 3
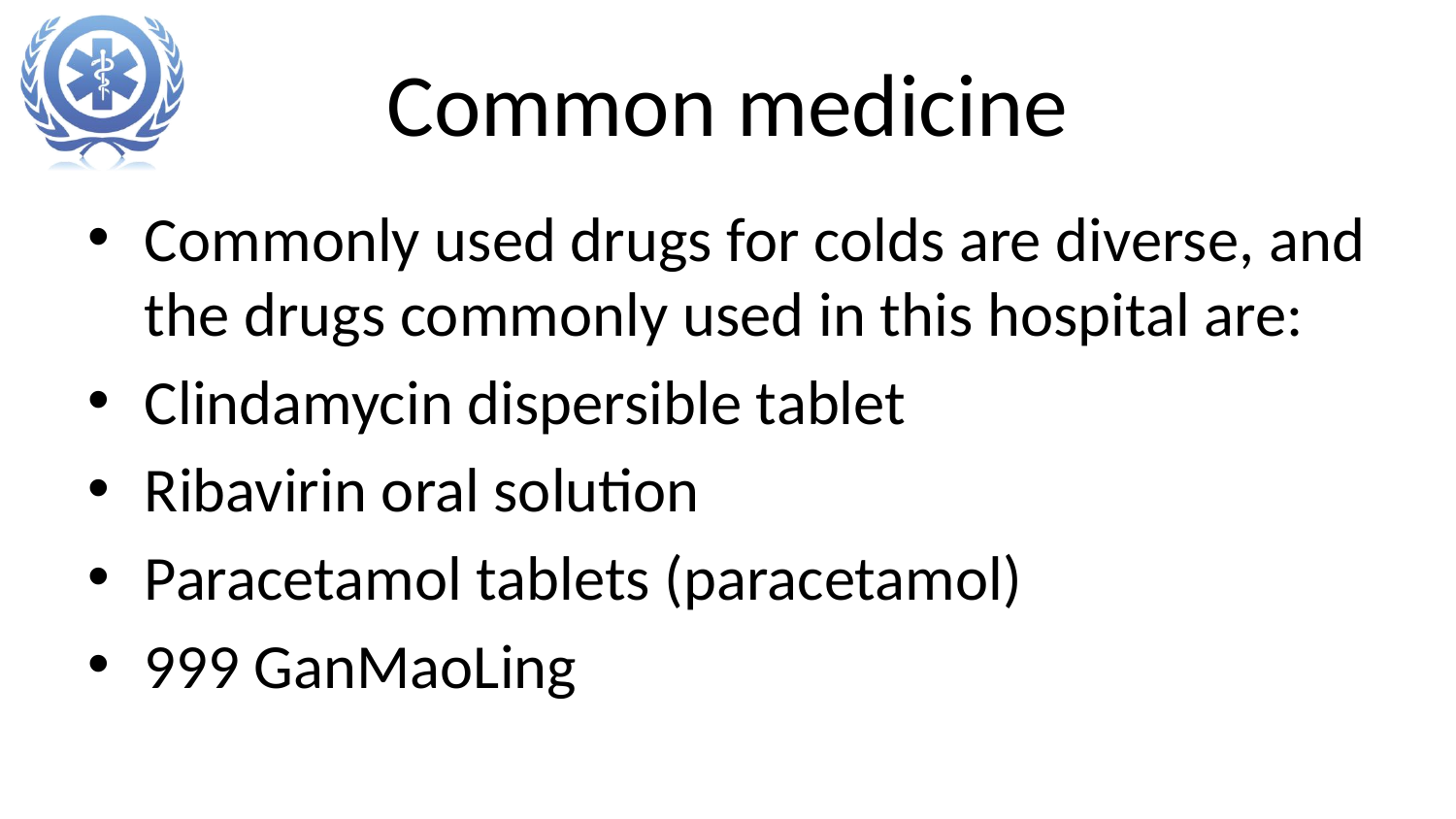

# Common medicine
Commonly used drugs for colds are diverse, and the drugs commonly used in this hospital are:
Clindamycin dispersible tablet
Ribavirin oral solution
Paracetamol tablets (paracetamol)
999 GanMaoLing

## Slide 4
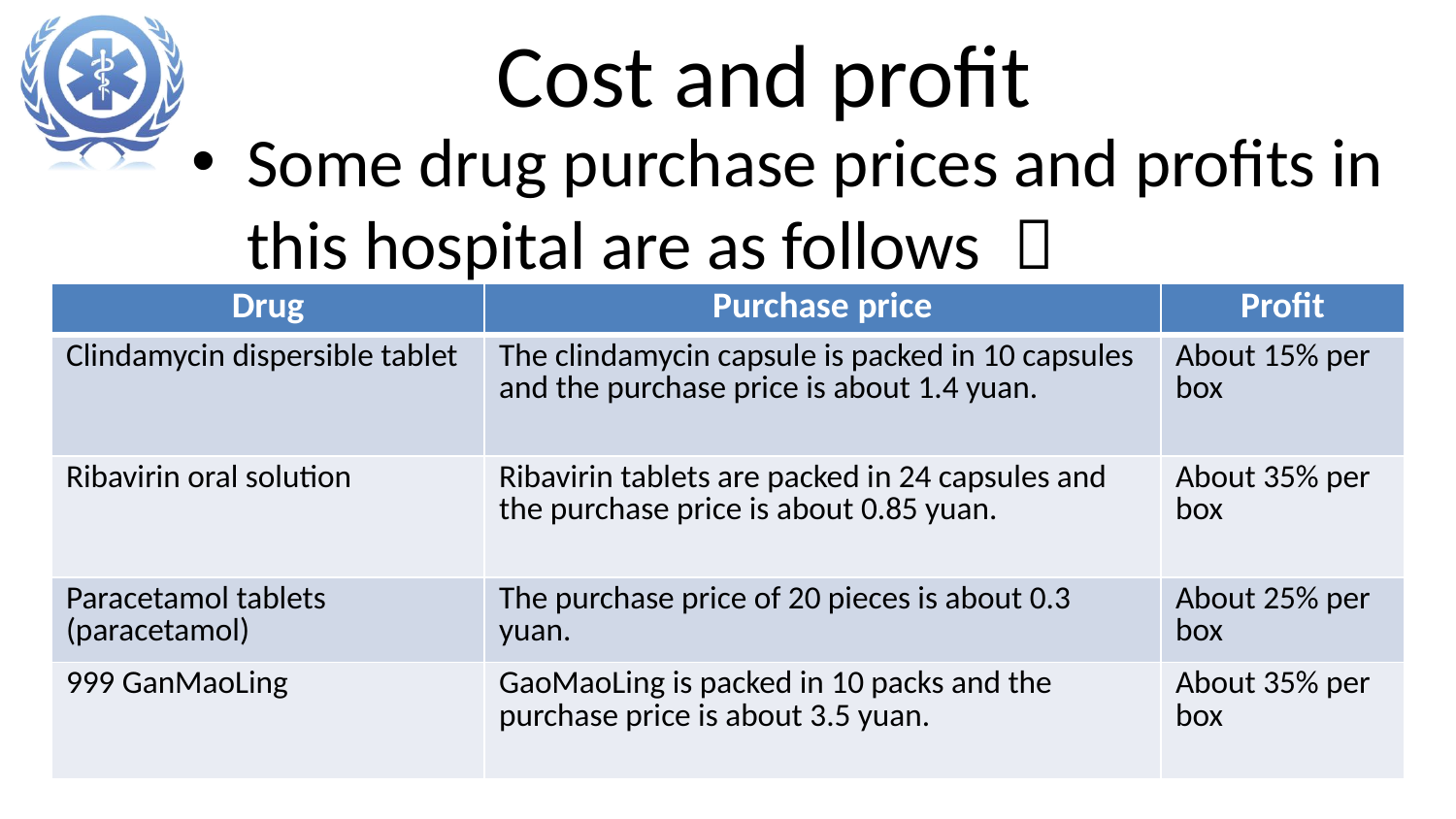

# Cost and profit
Some drug purchase prices and profits in this hospital are as follows ：
| Drug | Purchase price | Profit |
| --- | --- | --- |
| Clindamycin dispersible tablet | The clindamycin capsule is packed in 10 capsules and the purchase price is about 1.4 yuan. | About 15% per box |
| Ribavirin oral solution | Ribavirin tablets are packed in 24 capsules and the purchase price is about 0.85 yuan. | About 35% per box |
| Paracetamol tablets (paracetamol) | The purchase price of 20 pieces is about 0.3 yuan. | About 25% per box |
| 999 GanMaoLing | GaoMaoLing is packed in 10 packs and the purchase price is about 3.5 yuan. | About 35% per box |

## Slide 5
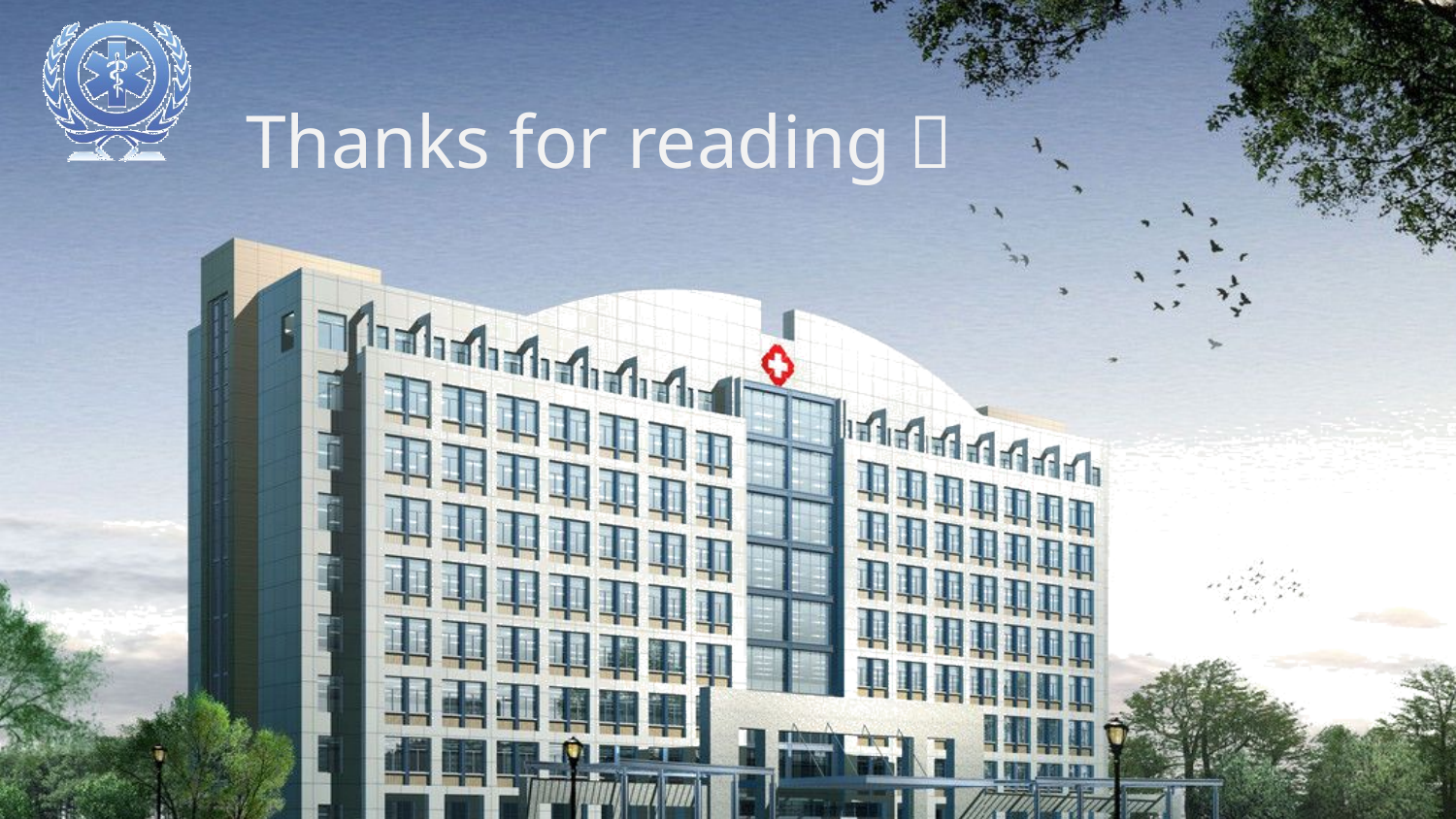

Thanks for reading！
